# Effect of ZnO and CuO nanoparticles on the growth, nutrient absorption, and potential health risk of the seasonal vegetable *Medicago polymorpha* L.

**DOI:** 10.7717/peerj.14038

**Published:** 2022-09-21

**Authors:** Hongting Ji, Zhi Guo, Guodong Wang, Xin Wang, Hongjiang Liu

**Affiliations:** 1 Jiangsu Academy of Agricultural Sciences, Nanjing Institute of Agricultural Sciences in Jiangsu Hilly Area, Nanjing, Jiangsu, China; 2 Jiangsu Academy of Agricultural Sciences, Institute of Agricultural Resources and Environment, Nanjing, Jiangsu, China

**Keywords:** Zinc oxide nanoparticles, Copper oxide nanoparticles, Growth performance, Nutrient absorption, Health risk assessment

## Abstract

**Background:**

*Medicago polymorpha* L., a seasonal vegetable, is commonly grown in China. The increasing use of nanoparticles (NPs) such as ZnO and CuO NPs in agriculture has raised concerns about their potential risks for plant growth and for human consumption. There is a lack of research on the effects of ZnO and CuO NPs on agronomic performance of* Medicago polymorpha* L. and their potential risks for human health.

**Methods:**

In this study, different treatment concentrations of ZnO NPs (25, 50, 100, and 200 mg kg^−1^) and CuO NPs (10, 25, 50, and 100 mg kg^−1^) were used to determine their effects on the growth and nutrient absorption of* Medicago polymorpha* L., as well as their potential risk for human health.

**Results:**

The results showed that ZnO and CuO NPs increased the fresh weight of *Medicago polymorpha* L. by 5.8–11.8 and 3.7–8.1%, respectively. The best performance for ZnO NPs occurred between 25–50 mg kg^−1^ and the best performance for CuO NPs occurred between 10–25 mg kg^−1^. Compared with the control, ZnO and CuO NPs improved the macronutrients phosphorus (P), potassium (K), magnesium (Mg), and calcium (Ca). The following micronutrients were also improved: iron (Fe), nickel (Ni), copper (Cu), zinc (Zn), and manganese (Mn), with the exception of nitrogen (N) accumulation. Low treatment concentrations exhibited more efficient nutrient uptake than high treatment concentrations. A comprehensive analysis showed that the optimum concentrations were 25 mg kg^−1^ for ZnO NPs and 10 mg kg^−1^ for CuO NPs. The potential non-carcinogenic health risk of *Medicago polymorpha* L. treated with ZnO and CuO NPs was analyzed according to the estimated daily intake (EDI), the hazard quotient (HQ), and the cumulative hazard quotient (CHQ). Compared with the oral reference dose, the EDI under different ZnO and CuO NPs treatments was lower. The HQ and CHQ under different ZnO and CuO NPs treatments were far below 1. This indicated that *Medicago polymorpha* L. treated with ZnO and CuO NPs did not pose any non-carcinogenic health risk to the human body. Therefore, ZnO and CuO NPs were considered as a safe nano fertilizer for *Medicago polymorpha* L. production according to growth analysis and a human health risk assessment.

## Introduction

Grass mulching in orchards is one of the most effective ways to resolve the many problems faced by orchards in China, such as single intensive planting, the simple structure of the biological community, and decline of species diversity. In addition, grass mulching in orchards impacts their ecology in various ways, including: the conservation of the soil and water, a reduction in nutrient emissions, improved soil quality and near-ground microenvironment, enhanced fruit quality, and pest and weed control (Wang et al., 2015; Jiao et al., 2017). Leguminous species (*Lolium perenne* L., *Medicago sativa* L., etc.) and gramineous species (*Dactylis glomerata* L., *Trifolium repens* L., etc.) are common grass species in orchards in China (Zhou et al., 2017a; Zhou et al., 2017b). *Medicago polymorpha* L. is an annual forage of leguminous alfalfa, which is native to the Mediterranean basin. It is also widely grown in Australia, Chile, South Africa, and the United Sates (Graziano et al., 2010). *Medicago polymorpha* L. is characterized by the high nutrient value, and is the high-quality feed for grazing livestock (Peck, Michelnore & Sutton, 2021). Furthermore, the leaves and plant-derivatives of *Medicago polymorpha* L. are consumed by humans. It is regarded as a seasonal vegetable in southern China (Chen et al., 2018). It is rich in nutrients, contains eighteen amino acids, and is abundant in vitamins (vitamin C, B_1_, B_2_, and E), and trace elements (copper (Cu), manganese (Mn), iron (Fe), zinc (Zn), and magnesium (Mg)) (Ma et al., 2015). Additionally, it is low in fat and high in protein and dietary fiber. It may also be used as a dietary supplement for people with obesity, diabetes, and hyperlipidemia (Chen et al., 2018).

Zn and Cu are primary heavy metal contaminants in Chinese soil, which are mainly caused by agricultural activities such as the application of fertilizers and pesticides (Chen et al., 2018). In order to ensure fruits yield and greater economic benefits, farmers have applied large quantities of fertilizers and pesticides to fruit trees, leading to the enrichment of Zn and Cu in soil and edible parts of plants (Wang & Li, 2014). ZnO and CuO nanoparticles (NPs) have been some of the most widely used NPs in agriculture (Hong et al., 2015; Yang et al., 2021). It is estimated that more than 200 metric tons of CuO NPs were produced in 2010, and the annual production of ZnO NPs was greater than 5,500 metric tons (Rajput et al., 2020). ZnO and CuO NPs have been used as a nano fertilizer (Elemike et al., 2019), and nano pesticide (nano insecticide, nano fungicide, and nano herbicide) in agriculture (Elmer & White, 2018). As a nano fertilizer, they may promote growth and nutrient uptake and increase the yield of crops (Elemike et al., 2019; Zhao et al., 2020). As a nano pesticide, ZnO and CuO NPs are the effective means to control crop disease and pests (Elmer & White, 2018). Therefore, there may be an exponentially-increasing use of ZnO and CuO NPs in agriculture in the future. Much of the literature has reported that ZnO and CuO NPs cause negative effects on plants and human health. Du et al. (2019) found that ZnO NPs negatively affected seed germination, root length, shoot length, and the dry biomass of wheat seedlings. ZnO NPs at 100, 200, and 1,000 mg L^−1^ significantly decreased the root length and shoot length by 67–77% and 13–18%, respectively. ZnO NPs at 1,000 mg L^−1^ significantly decreased seed germination and the total dry weight. Rui et al. (2018) reported that CuO NPs at 500 mg kg^−1^ significantly reduced the fresh shoot biomass and 1,000-grain weight of peanut. In addition, exposure to 50 and 500 mg kg^−1^ CuO NPs resulted in 36.2 and 21.1% decreases in the total amino acid content, respectively (Rui et al., 2018). Deng et al. (2022) reported that CuO NPs at 75 mg kg^−1^ significantly decreased the grain yield of cultivated rice by 38.3%, and CuO NPs at ≥300 mg kg^−1^ caused no grain production of rice. More alarmingly, Zn and Cu released by ZnO and CuO NPs may be enriched in the edible part of crops, which may threaten human health through their consumption (Rajput et al., 2020). Thus, it is necessary to assess the negative effects of ZnO and CuO NPs on the growth performance of plants and human health.

Due to the importance of food security, several studies have investigated the impacts of ZnO and CuO NPs on the major grain crops (*e.g*., wheat, rice, and maize) (Dimkpa et al., 2017; Du et al., 2019; Sun et al., 2020; Yang et al., 2021). It should be noted that, in these studies, it was common for the NPs to be directly applied to the edible tissue of vegetables. However, there are fewer studies on vegetables than on grain crops, and the results are inconsistent. For example, Deng et al. (2020) reported that CuO NPs (75, 150, 300, and 600 mg kg^−1^) significantly affected the potassium (K), phosphorus (P), Fe, Zn, Cu of bok choy, but had no significant effect on their boron (B), calcium (Ca), Mg, Mn, molybdenum (Mo), and sulfur (S) contents. Zuverza-Mena et al. (2015) reported that CuO NPs (20 and 80 mg kg^−1^) significantly reduced shoot elongation and relative chlorophyll content and decreased the macroelements and microelements B, Zn, Mn, Ca, P, and S in the shoots of cilantro. However, Wang et al. (2020) found that CuO NPs (150 mg kg^−1^) increased root Ca, root Fe, bulb Ca, and the bulb Mg of green onion. The differences in results may be associated with the species of vegetables and the concentration of the treatments (Deng et al., 2020; Hong et al., 2015; Tan et al., 2018).

To our knowledge, there has been no study to date on the effects of ZnO and CuO NPs on *Medicago polymorpha* L. Cota-Ruiz et al. (2020) reported the effects of Cu NPs on alfalfa (*Medicago sativa* L.). It should be noted that the effect of CuO NPs on plant growth and nutrient uptake was different from that of Cu NPs (Hong et al., 2015). In addition, the response of plants to NPs was species-dependent (Deng et al., 2022; Tan et al., 2018). Although *Medicago sativa* L. and *Medicago polymorpha* L. are classified to the same genus, they are different species (Ren, Wei & Chen, 2014). It is unknown whether ZnO and CuO NPs have negative effects on the growth of *Medicago polymorpha* L. Furthermore, leafy vegetables may have a greater potential for accumulating heavy metal microelements in their edible parts versus grain crops due to the direct application of NPs on the edible tissue of vegetables. The edible organs of *Medicago polymorpha* L. plants may enrich Zn and Cu under ZnO and CuO NPs application. However, whether ZnO and CuO NPs was hazardous to humans requires further investigation. The United States Environmental Protection Agency (USEPA) provided guidelines on the estimated daily intake (EDI) of metal exposure by humans (United States Environmental Protection Agency, 1989). The hazard quotient (HQ) and cumulative HQ (CHQ) were widely used to represent the non-carcinogenic health risk effect of metal elements (Natasha et al., 2020; Singh & Kumar, 2020; Shen et al., 2022; Wang et al., 2022).

Thus, this study aimed to investigate the agronomic performances of *Medicago polymorpha* L. under ZnO and CuO NPs application, which included the growth parameters and the accumulation of macroelements and microelements. Correlation analysis and membership function methods were used to comprehensively evaluate the effects of ZnO and CuO NPs on these parameters. Furthermore, the EDI, HQ and CHQ were used to assess the non-carcinogenic health risk of *Medicago polymorpha* L. treated with ZnO and CuO NPs.

## Material and Methods

### The properties of ZnO NPs and CuO NPs

ZnO and CuO NPs were purchased from Shanghai Macklin Biochemical Co., Ltd., Shanghai, China, and Sigma-Aldrich Chemistry (St. Louis, MO, USA), respectively. ZnSO_4_ ⋅ 7H_2_O and CuSO_4_ ⋅ 5H_2_O were purchased from Xilong Chemical Reagent Co., Ltd., (Shantou, China), and Nanjing Chemical Reagent Co., Ltd., (Nanjing, China). The properties of ZnO and CuO NPs are shown in Table 1.

### Experimental design

There were seventeen treatments including the control, ZnSO_4_ at the concentrations of 25, 50, 100, 200 mg kg^−1^, ZnO NPs at the concentrations of 25, 50, 100, 200 mg kg^−1^, CuSO_4_ at the concentrations of 10, 25, 50, 100 mg kg^−1^, and CuO NPs at the concentrations of 10, 25, 50, 100 mg kg^−1^, respectively. These treatment concentrations were selected according to the soil environmental quality standard (Ministry of Ecology and Environment of China, 2018) and our measured Zn (∼30 mg kg^−1^) and Cu (∼20 mg kg^−1^) concentrations of the soil in the Experimental Station of Plant Science of Jiangsu Academy of Agricultural Science (31°36′N, 119°11′E). The environmental quality standard value of Zn and Cu were 250 and 100 mg kg^−1^ in soils with the pH of 6.5−7.5, respectively (Ministry of Ecology and Environment of China, 2018). The no trace element (Zn and Cu) application treatment was used as the control. The experimental cultivar was *Medicago polymorpha* L., and the seeds were purchased from Wenling Shennong Seed Co., Ltd., (Wenling, China). A total of 2 g of seeds were sown into a plastic cup filled with 110 g vegetable seedling substrate, and irrigated with 100 ml deionized water, with a subsequent covering with 5 g vegetable seedling substrate. The seedlings were thinned to 12 plants per cup ten days after emergence. The seedlings were cultured in a growth chamber (RDN-1000G-2, Ningbo Dongnan Instrument Co., Ltd.; Ningbo, China). The maintained parameters during the plant growth period were set as follows: 22/20 °C day/night with a 16/8 h light/dark cycle with a light intensity of 42,000 lx and 50% relative humidity. The substrate was the “YouJia” vegetable seedling substrate, purchased from Zhongyuan Horticulture Development Co., Ltd. (Huai’an, China). The contents of nitrogen (N), P, K, and organic matter of the substrate were 12.8, 3.91, 19.2, and 406.9 mg g^−1^, respectively. The N, P, and K contents were measured with the methods described in our previous study (Yang et al., 2021). The organic matter content was measured with the potassium dichromate-volumetric method (Xu et al., 2016). ZnSO_4_ and CuSO_4_ were fully dissolved with deionized water and were prepared to the same concentration as the ZnO and CuO NPs. An NPs suspension or metal ions solution was applied to the substrate five days after the seedlings were fixing. The exposure duration was 15 days. A total of 4.14 g urea (*N* = 46%) and 2.11 g compound fertilizer (N-P-K = 15%–15%-15%) were added to each cup after being dissolved in water. The fertilizers were applied every five days after the seedlings were thinned and the treatments were applied three times in total.

**Table 1:** The properties of ZnO and CuO NPs used in this study.

Properties	ZnO NPs	CuO NPs
Particle size (nm)	30 ± 10	<50
Purity (%)	>99.9%	>99.5%
Average hydrodynamic (nm)	124.7 ± 8.9	500.1 ± 7.3
Average zeta potential (mV)	23.8 ± 1.6	−31.9 ± 1.5

### Measurements and analysis

#### Fresh weight

Five to six cups of plants were sampled to weigh the fresh shoot weight of *Medicago polymorpha* L for each treatment.

### The concentration of macronutrients and micronutrients

The samples were manually ground into fine powders using a mortar. The powders were added to a digest tube containing 25 mL sulfuric acid (98%) and one mL hydrogen peroxide (30%). The concentrations of N, P, and K were measured with the methods used in our previous study (Yang et al., 2021). The concentrations of Fe, Ni, Zn, Cu, Mn, Mg, and Ca were determined by the inductively coupled plasma mass spectrometry (ICP-MS) (NexION 2000; PerkinElmer, Fremont, CA, USA).

### Human health risk assessment

The estimated daily intake (EDI), hazard quotient (HQ), and cumulative HQ (CHQ) were calculated to assess the potential non-carcinogenic health risk of *Medicago polymorpha* L. treated with ZnO and CuO NPs. The EDI of the metal element was calculated according to the method of the US Environmental Protection Agency (USEPA, 1989). The HQ and CHQ of the metal elements were calculated according to the methods of Zafeiraki et al. (2021) and Shen et al. (2022).

(1) \begin{eqnarray*}EDI= \frac{{C}_{ME}\times DI\times IF\times (EA-AA)}{BW\times AID} \end{eqnarray*}

(2) \begin{eqnarray*}HQ= \frac{EDI}{RfD} \end{eqnarray*}

(3) \begin{eqnarray*}CHQ=\sum _{i=1}^{n}H{Q}_{i}\end{eqnarray*}

*EDI* (mg kg^−1^ d^−1^) was the index of estimated daily intake of metal element. *C*_*ME*_ (mg kg^−1^) was the metal element concentration of *Medicago polymorpha* L. *DI* (kg d^−1^) was the daily intake of *Medicago polymorpha* L. *IF* (d a^−1^) was the intake days per year. The values of *DI* and *IF* were obtained through investigation in southern Jiangsu. The values of *DI* were 0.10 kg d^−1^ for adult males, 0.08 kg d^−1^ for adult females, 0.02 kg d^−1^ for young children (<6 years old), respectively. The values of *IF* were 70 d a^−1^ for adults and 50 d a^−1^ for young children, respectively. *EA* (a) and *AA* (a) were the expected age and the average age for adults and young children, respectively. *BW* (kg) and *AID* (d) were average body weight and average total intake days. The values of *EA*, *AA*, *BW,* and *AID* were referred from the data reported by Yang et al. (2021). *HQ* represented the non-carcinogenic risk effect. *RfD* (mg kg^−1^d^−1^) was the oral reference dose of daily intake, and the values of *RfD* for Fe, nickel (Ni), Zn, Cu, Mn were 0.70, 0.02, 0.30, 0.037, 0.24 mg kg^−1^ d^−1^ (Kicinska, Glichowska & Mamak, 2019; Shen et al., 2022; Zhu et al., 2011; Wang et al., 2022). A value of *HQ* ≤1 indicated that Zn and Cu treated *Medicago polymorpha* L. had no non-carcinogenic risk to human health. A value of *HQ* > 1 indicated that Zn and Cu treated *Medicago polymorpha* L. had a certain non-carcinogenic risk to human health. A value of *HQ* >  10 indicated that Zn and Cu treated *Medicago polymorpha* L. had a high non-carcinogenic risk to human health (Zhou et al., 2017a; Zhou et al., 2017b). C*HQ* represented the effects of the cumulative non-carcinogenic risk of multi-metal elements.

### The comprehensive score under Zn and Cu treatments

The comprehensive score was calculated with reference to Yang & Yao (2009).

(4) \begin{eqnarray*}{D}_{i}& =\sum {U}_{ij}\times {W}_{j}\end{eqnarray*}

(5) \begin{eqnarray*}{W}_{j}& = \frac{\overline{{r}_{i}}}{\sum \overline{{r}_{i}}} \end{eqnarray*}

(6) \begin{eqnarray*}{U}_{ij}& = \frac{{X}_{ij}-{X}_{\mathrm{min}}}{{X}_{\mathrm{max}}-{X}_{\mathrm{min}}} .\end{eqnarray*}

*D*_*i*_ was the comprehensive score of the *i*th treatment. *W*_*j*_ was the weight value of the *j*th index. $\bar {{r}_{i}}$ was the average value of the correlation coefficient between the *j*th index and the other indexes. *U*_*ij*_ was the membership function value of the *j*th index under the *i*-th treatment. *X*_*ij*_ was the value of the *j*th index under the *i*th treatment. *X*_min_ was the minimum value of the *j*th index, and *X*_max_ was the maximum value of the *j*th index.

### Statistical analysis

Statistical analysis was carried out using SPSS statistical software (SPSS 20.0). The experimental data were analyzed using analysis of variance (ANOVA) to evaluate the main effects of metal forms, metal treatment concentration and their interactions on the fresh weight per plant and the accumulation of macronutrients and micronutrients. The least significant difference (*LSD*) test was used to determine the significance of differences between means at the 0.05 level. The *p* < 0.05 indicated that there were significant differences between different treatments. The *p* >  0.05 indicated that there were no significant differences between different treatments

## Results

### Biomass production of *Medicago polymorpha* L.

**Figure 1:**
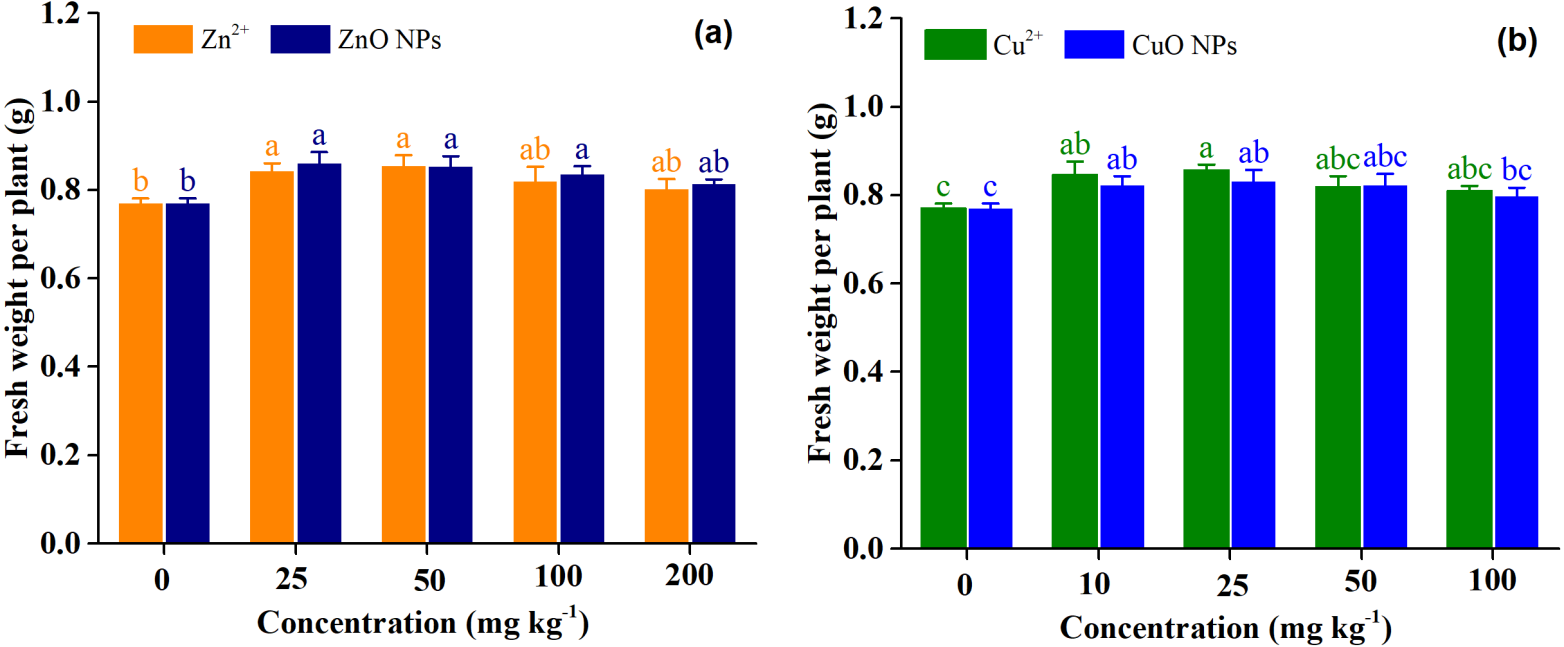
Fresh weight per plant of *Medicago polymorpha* L. under different Zn (A) and Cu (B) treatments. Different lowercase letters labeled on each bar represent the significant differences at 0.05 level within the metal ions and metal oxide nanoparticles treatments. Vertical bars represent standard error of mean.

Figure 1 shows the responses of fresh weight per plant (FWPP) of *Medicago polymorpha* L. to Zn treatments and Cu treatments. Overall, the effect of the ZnO (*p* = 0.51) and CuO NPs (*p* = 0.39) on FWPP was not significantly different from those of Zn^2+^ and Cu^2+^ when averaged across treatment concentrations (Table 2). This indicated that ZnO and CuO NPs did not have the negative effect on the FWPP compared with Zn^2+^ and Cu^2+^. Zn treatment concentrations (*p* = 0.002) and Cu treatment concentrations (*p* = 0.009) had a significant effect on FWPP (Table 2). Zn^2+^ and ZnO NPs treatments increased the FWPP, and Zn^2+^ and ZnO NPs exhibited higher efficiency for growth promotion at low concentrations than at high concentrations. The highest FWPP were obtained under Zn^2+^ and ZnO NPs at 25 mg kg^−1^. Compared with the control, Zn^2+^ treatments at the concentrations of 25, 50, 100, and 200 mg kg^−1^ significantly increased the FWPP by 9.6, 11.0, 6.5, and 4.3%, respectively. Compared with the control, ZnO NPs treatments at the concentrations of 25, 50, 100, and 200 mg kg^−1^ significantly increased the FWPP by 11.8, 10.8, 8.6, and 5.8%, respectively (Fig. 1A). Cu^2+^ and CuO NPs treatments increased the FWPP, which was similar to the results of the Zn treatments. Cu^2+^ and CuO NPs at 25 mg kg^−1^ produced the highest FWPP. High concentrations of Cu^2+^ and CuO NPs (50 and 100 mg kg^−1^) slightly increased the FWPP by 6.4 and 5.1, and 6.9 and 3.7%, respectively, but low concentrations of Cu^2+^ and CuO NPs (10 and 25 mg kg^−1^) significantly increased the FWPP by 10.0 and 11.2, and 7.0 and 8.1%, respectively, as compared with the control (Fig. 1B).

**Table 2:** Probability values of main effects of metal from, treatment concentration, and their interaction on growth and nutrients uptake under different Zn treatments and Cu treatments.

Treatment	Source of variation	FWPP	N	P	K	Mg	Ca	Fe	Ni	Zn	Cu	Mn
Zn	Zn form	0.51	0.04	0.39	0.14	0.50	0.76	0.004	0.16	0.72	0.18	0.02
	Treatment concentration	0.002	0.007	<0.001	<0.001	<0.001	0.004	<0.001	<0.001	<0.001	<0.001	<0.001
	Zn form & treatment concentration	0.98	0.57	0.31	0.75	0.81	0.67	0.11	0.76	0.75	0.02	0.07
Cu	Cu form	0.39	0.90	0.03	0.002	0.12	0.30	0.001	0.93	0.22	0.005	0.03
	Treatment concentration	0.009	0.03	<0.001	<0.001	<0.001	0.02	<0.001	<0.001	<0.001	<0.001	<0.001
	Cu form & treatment concentration	0.93	0.87	0.001	0.12	0.78	0.80	<0.001	0.09	0.24	0.52	0.20

### The accumulation of macronutrients under Zn and Cu treatments

**Figure 2:**
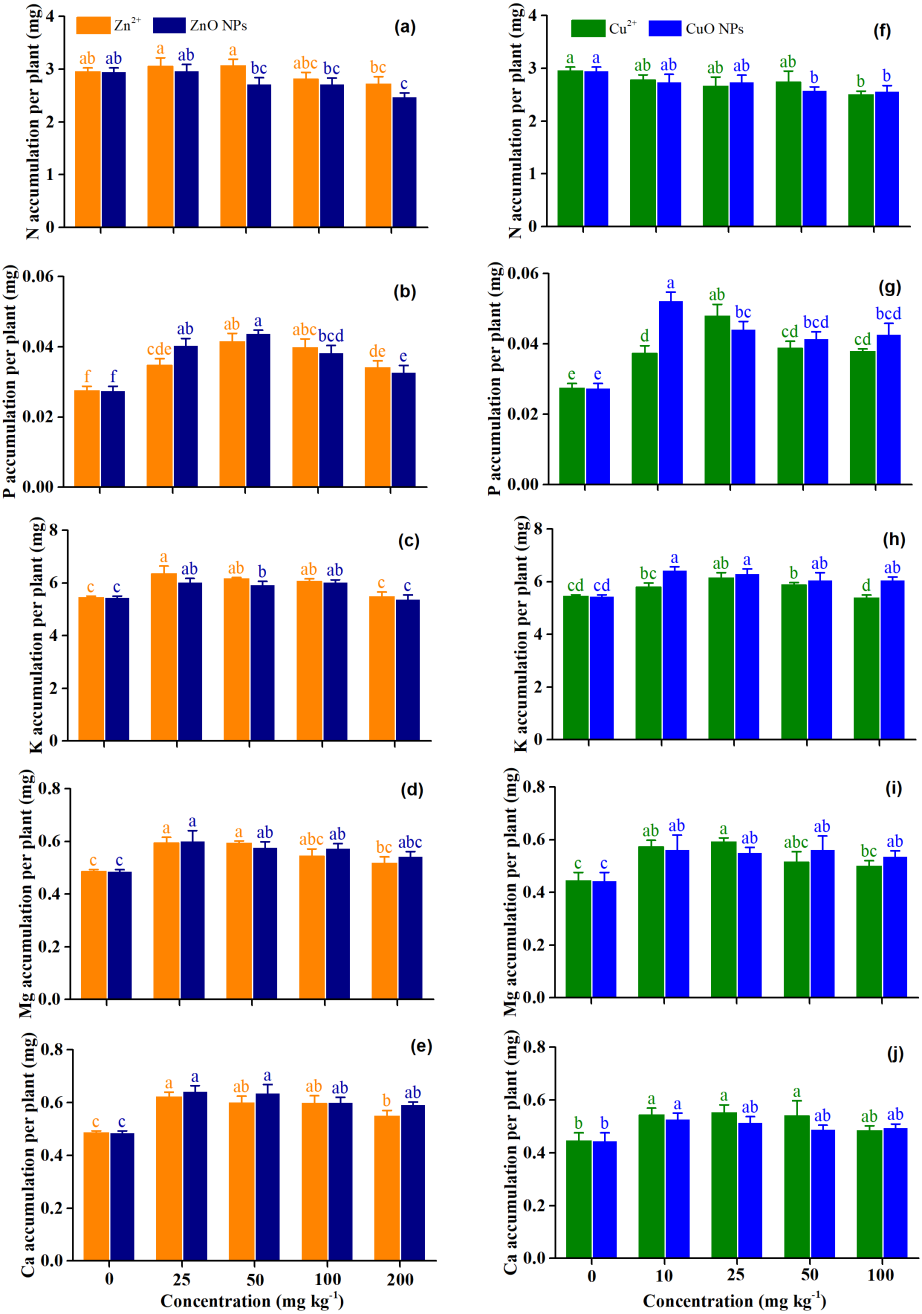
(A–J) The accumulation of macronutrients of* Medicago Polymorpha* L. plants under different Zn and Cu treatments. Different lowercase letters labeled on each bar represent the significant differences at 0.05 level within the metal ions and metal oxide nanoparticles treatments. Vertical bars represent standard error of mean.

Figure 2 shows the effects of Zn and Cu treatments on the accumulation of macronutrients (N, P, K, Mg, and Ca) of *Medicago polymorpha* L. The form of Zn significantly affected the accumulation of N (*p* = 0.04), and N accumulation under Zn^2+^ was 21.6% higher than that of ZnO NPs (Table 2 and Fig. 2A). The largest reduction was observed under ZnO NPs at 50 mg kg^−1^, which was 11.4% lower than for Zn^2+^ at 50 mg kg^−1^ (Fig. 2A). However, CuO NPs did not significantly affect the accumulation of N compared with the Cu^2+^ (*p* = 0.90) (Table 2 and Fig. 2F). Zn^2+^ treatment concentrations had no significant effect on the accumulation of N (*p* >  0.05), although Zn^2+^ at concentrations of 100 and 200 mg kg^−1^ decreased the accumulation of N by 4.8 and 8.2%, respectively. There was no significant effect of ZnO NPs on the accumulation of N at concentrations of 25–100 mg kg^−1^ (*p* >  0.05). However, ZnO NPs at concentrations of 200 mg kg^−1^ significantly decreased the accumulation of N by16.2% (*p* < 0.05) (Fig. 2A). For Cu treatments, Cu^2+^ at concentrations of 100 mg kg^−1^ and CuO NPs at concentrations of 50–100 mg kg^−1^ significantly decreased the N accumulation by 15.5 and 12.8–13.2%, respectively (*p* < 0.05). However, there was no significant difference between other treatments and the control (Fig. 2F).

ZnO NPs had no significant effect on the P accumulation compared with Zn^2+^ when averaged across treatment concentrations (*p* = 0.39). Zn treatment concentrations significantly increased the P accumulation of plants (*p* < 0.001) when averaged across Zn forms (Table 2). Compared with the control, the P accumulation was significantly increased by 23.7–50.9% under Zn^2+^ treatments, by 19.2–59.2% under ZnO NPs treatments. The highest P accumulation were observed under Zn^2+^ and ZnO NPs at 50 mg kg^−1^ (Fig. 2B). The P accumulation of the CuO NPs was 11.6% higher than that of the Cu^2+^ when averaged across treatment concentrations (*p* = 0.03). Cu treatment concentrations significantly increased the accumulation of P by 36.0–74.2% under Cu^2+^ treatments, and by 49.1–90.1% under CuO NPs treatment (*p* < 0.001), and the effect of CuO NPs on the accumulation of P was higher than that of Cu^2+^ treatments (*p* = 0.03). The Cu^2+^ at 25 mg kg^−1^ and CuO NPs at 10 mg kg^−1^ produced the highest P accumulation (Table 2 and Fig. 2G).

Zn treatment concentrations significantly increased the K accumulation of plants except for Zn^2+^ and ZnO NPs at 200 mg kg^−1^ when compared with the control (*p* <0.05). The highest K accumulation was obtained with Zn^2+^ and ZnO NPs at 25 mg kg^−1^ (Fig. 2C). Cu treatment concentrations significantly affected the accumulation of K (*p* < 0.001) (Table 2). The highest K accumulation was obtained with Cu^2+^ 25 mg kg^−1^ andCuO NPs at 10 mg kg^−1^. Cu^2+^ at 25 and 50 mg kg^−1^ significantly increased K accumulation by 12.6 and 7.1%, respectively (*p* < 0.05). Both Cu^2+^ at 10 and 100 kg^−1^ did not affect the K accumulation (*p* > 0.05). CuO NPs treatments significantly increased the K accumulation by 11.2–18.1% (*p* < 0.05), and K accumulation decreased with the increasing concentrations of CuO NPs (Fig. 2H). No significant difference in K accumulation was observed between Zn^2+^ and ZnO NPs (*p* = 0.14) when averaged across treatment concentrations (Table 2). However, CuO NPs produced a higher K accumulation than for Cu^2+^ (*p* = 0.002) (Table 2).

Both Zn and Cu treatment concentrations significantly affected the accumulation of Mg (*p* < 0.001 and *p* <0.001) and Ca (*p* = 0.004 and *p* = 0.02) when averaged across metal forms. ZnO and CuO NPs had no significant effect on the accumulation Mg and Ca compared with their ionic treatments (*p* > 0.05) (Table 2). Compared with the control, the accumulation of Mg and Ca increased by 6.5–22.4% and 12.2–33.0% under Zn^2+^ treatment concentrations, and by 11.9–23.8% and 20.6–26.4% under ZnO NPs treatment concentrations. Both Zn^2+^ and ZnO NPs at 25 mg kg^−1^ produced the highest Mg and Ca accumulations (Figs. 2D, and 2E). Compared with the control, the accumulation of Mg and Ca increased by 13.0–28.1% and 8.9–24.1% under Cu^2+^ treatment concentrations, and by 21.8–32.4% and 9.9–18.5% under CuO NPs treatment concentrations. Both Cu^2+^ at 25 mg kg^−1^ and CuO NPs at 10 mg kg^−1^ produced the highest Mg accumulation and Ca accumulation (Figs. 2I and 2J).

### The accumulation of micronutrients under Zn and Cu treatments

**Figure 3:**
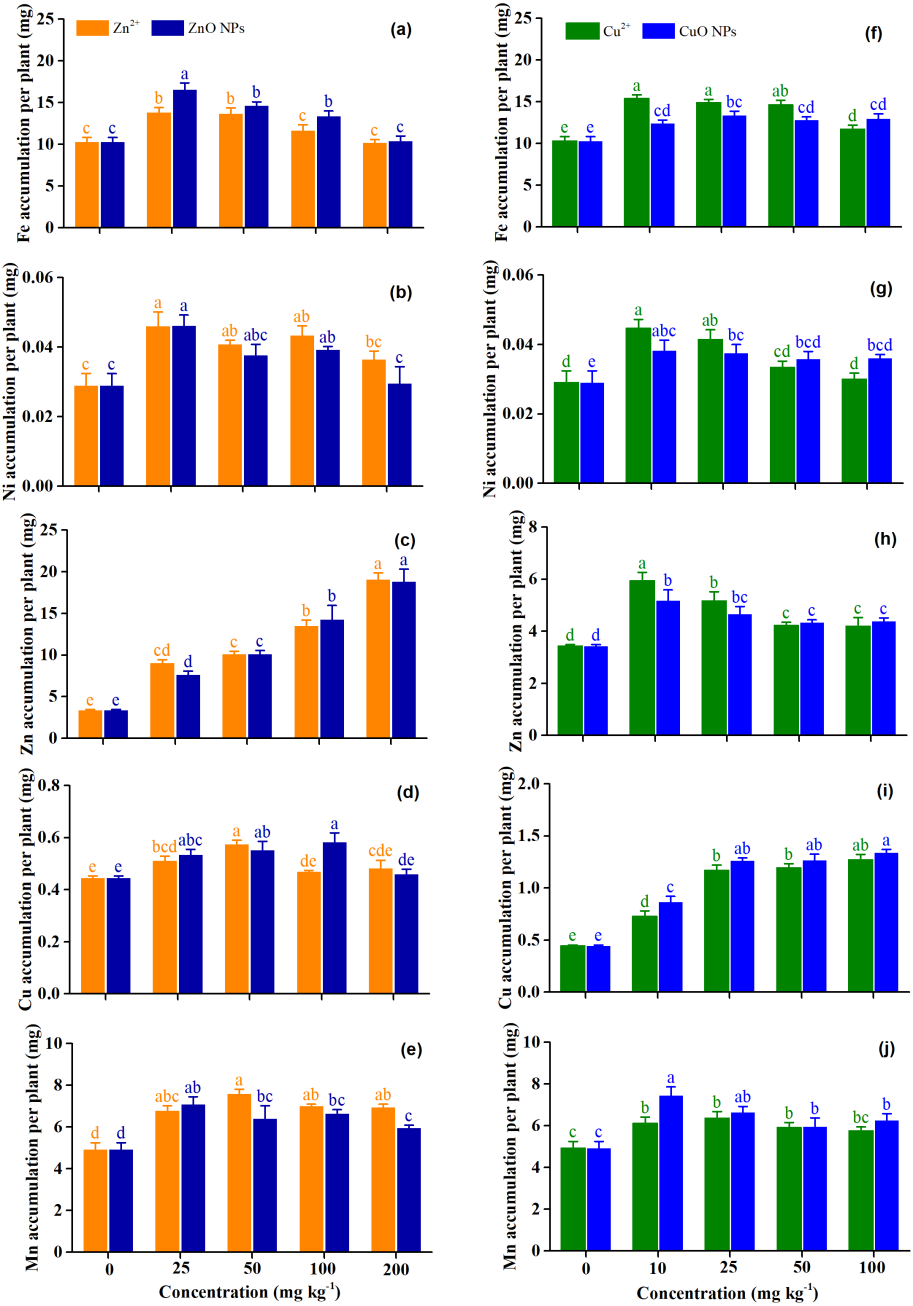
(A–J) The concentration of micronutrients of* Medicago Polymorpha* L. plants under different Zn and Cu treatments. Different lowercase letters labeled on each bar represent the significant differences at 0.05 level within the metal ions and metal oxide nanoparticles treatments. Vertical bars represent standard error of mean.

Figure 3 shows the accumulation of micronutrients (Fe, Ni, Zn, Cu, and Mn) under Zn and Cu treatments. The Fe accumulation under ZnO NPs was 11.2% higher than that of Zn^2+^ (*p* = 0.004). Zn treatment concentrations significantly affect the accumulation of Fe (*p* = 0.001) and Ni (*p* < 0.001) (Table 2). The highest accumulation of Fe and Ni were observed under the Zn^2+^ at 25 mg kg^−1^ and ZnO NPs at 25 mg kg^−1^, and the accumulation of Fe and Ni decreased with the increasing Zn^2+^ and ZnO NPs (Figs. 3A and 3B). The application of Zn^2+^ and ZnO NPs significantly enhanced the Zn accumulation of plants, as expected, and with the increasing concentration of Zn^2+^ and ZnO NPs, the Zn concentration of plants increased (Fig. 3C). The effect of Zn treatment concentrations (*p* < 0.001) and the interaction effect of Zn forms and Zn treatment concentrations (*p* = 0.02) on the accumulation of Cu were significant. However, the effect of Zn forms on the accumulation of Cu was not significant (*p* = 0.18) (Table 2). Compared with the control, Zn^2+^ at 25–50 mg kg^−1^ significantly increased the accumulation of Cu by 14.8–29.0%, and ZnO NPs at 25–100 mg kg^−1^ significantly increased the accumulation of Cu by 19.9–30.6%. The highest Cu accumulation was obtained with the Zn^2+^ 50 mg kg^−1^ andZnO NPs at 100 mg kg^−1^. Cu accumulation tended to increase under Zn^2+^ at 100–200 mg kg^−1^ and ZnO NPs at 200 mg kg^−1^, although the effect of treatment concentrations was not significant (Fig. 3D). The Mn accumulation of ZnO NPs was 7.8% lower than that of Zn^2+^ (*p* = 0.02). Zn treatment concentrations increased the accumulation of Mn by 16.7–28.7% when averaged across the Zn forms. The highest Mn accumulation was obtained with the Zn^2+^ 50 mg kg^−1^ andZnO NPs at 25 mg kg^−1^ (Fig. 3E).

Cu treatment concentrations significantly affected the accumulation of micronutrients (*p* < 0.001) (Table 2). Cu^2+^ and CuO NPs at 10–100 mg kg^−1^ significantly increased the accumulation of Fe by 13.5–49.3 and 20.4–29.6%, respectively. The highest Fe accumulation was observed for Cu^2+^ at 10 mg kg^−1^ and CuO NPs at 25 mg kg^−1^ (Fig. 3F). Low concentration of Cu treatments significantly increased the accumulation of Ni and Zn (*p* < 0.05), while the high concentration of Cu treatments had no significant effect on the accumulation of Ni and Zn (*p* > 0.05) (Fig. 3H). As expected, all of the Cu treatments increased the accumulation of Cu, and the Cu accumulation was higher under CuO NPs than for Cu^2+^. At 10 mg kg^−1^, the Cu accumulation was significantly higher for CuO NPs than for Cu^2+^ (*p* < 0.05). The Mn accumulation of CuO NPs was 8.9% higher than that of Cu^2+^ when averaged across Cu treatment concentrations (*p* = 0.03). Compared with the control, Cu^2+^ at 10–50 mg kg^−1^ and CuO NPs at 10–100 mg kg^−1^ significantly increased the accumulation of Mn by 19.8–28.7%, and 20.7–50.9%, respectively (*p* < 0.05). The highest Mn accumulation was observed for Cu^2+^ at 25 mg kg^−1^ and CuO NPs at 10 mg kg^−1^ (Fig. 3J).

**Figure 4:**
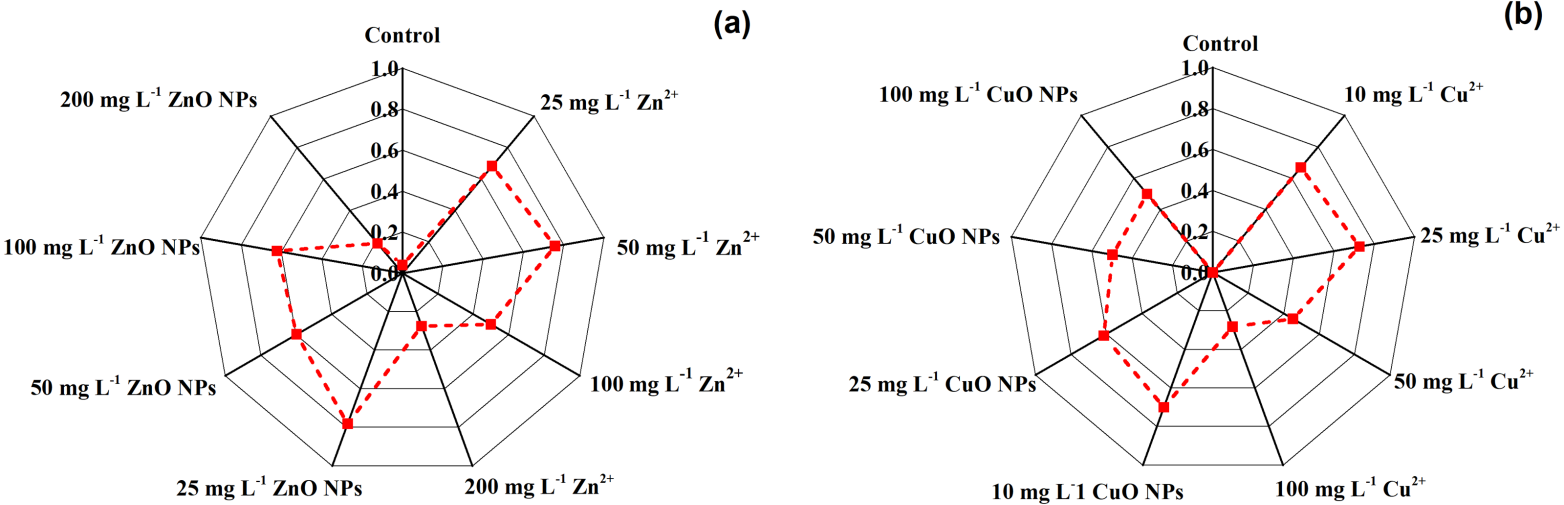
The comprehensive score of different Zn treatments (A) and Cu treatments (B).

The comprehensive effects of Zn and Cu treatments were summarized by the correlation analysis and membership function method. Table 3 shows the correlation coefficient, average coefficient, and weight values of evaluation indices. Figure 4 shows the comprehensive score of different Zn and Cu treatments. The Zn and Cu treatments had higher comprehensive scores than for the control. For Zn^2+^ and Cu^2+^ treatments, the highest comprehensive score was observed at the concentration of 50 mg kg^−1^ and 25 mg kg^−1^. For ZnO NPs and CuO NPs treatments, the highest comprehensive score was observed at the concentration of 25 mg kg^−1^ and 10 mg kg^−1^ (Fig. 4).

**Table 3:** Correlation coefficient, averaged correlation coefficient and weight values of evaluation indices.

Treatment		FWPP	N	P	K	Fe	Ni	Cu	Zn	Mg	Mn	Ca	$\bar {r}$	Weight value
Zn	FWPP	1											0.673	0.121
	N	0.2	1										0.202	0.036
	P	0.85^∗∗^	0.04	1									0.560	0.101
	K	0.71^∗^	0.64	0.56	1								0.594	0.107
	Fe	0.89^∗∗^	0.39	0.71^∗^	0.69^∗^	1							0.576	0.104
	Ni	0.69^∗^	0.53	0.53	0.90^∗∗^	0.68^∗^	1						0.592	0.107
	Cu	0.80^∗∗^	0.2	0.73^∗^	0.6	0.74^∗^	0.52	1					0.565	0.102
	Zn	−0.05	−0.72^∗^	0.03	−0.3	−0.42	−0.1	−0.09	1				−0.135	−0.024
	Mg	0.98^∗∗^	0.29	0.74^∗^	0.78^∗^	0.87^∗∗^	0.74^∗^	0.77^∗^	−0.09	1			0.675	0.122
	Mn	0.74^∗^	0.28	0.72^∗^	0.66	0.5	0.79^∗^	0.59	0.28	0.71^∗^	1		0.599	0.108
	Ca	0.93^∗∗^	0.17	0.70^∗^	0.71^∗^	0.72^∗^	0.64	0.79^∗^	0.11	0.96^∗∗^	0.74^∗^	1	0.647	0.117
Cu	FWPP	1											0.549	0.099
	N	−0.57	1										−0.278	−0.050
	P	0.58	−0.53	1									0.499	0.090
	K	0.47	−0.15	0.81^∗∗^	1								0.507	0.091
	Fe	0.84^∗∗^	−0.43	0.37	0.37	1							0.490	0.088
	Ni	0.81^∗∗^	−0.35	0.5	0.58	0.78^∗^	1						0.535	0.096
	Cu	0.3	−0.69^∗^	0.47	0.35	0.29	0.04	1					0.170	0.031
	Zn	0.85^∗∗^	−0.38	0.54	0.45	0.75^∗^	0.93^∗∗^	−0.01	1				0.532	0.096
	Mg	0.76^∗^	−0.3	0.76^∗^	0.82^∗∗^	0.67^∗^	0.75^∗^	0.4	0.76^∗^	1			0.626	0.113
	Mn	0.56	−0.25	0.90^∗∗^	0.85^∗∗^	0.35	0.57	0.3	0.64	0.88^∗∗^	1		0.542	0.098
	Ca	0.87^∗∗^	−0.4	0.61	0.51	0.91^∗∗^	0.74^∗^	0.24	0.80^∗∗^	0.77^∗^	0.61	1	0.567	0.102

**Notes.**

$\bar {r}$ indicated that the average value of correlation coefficient of evaluation indexes. One asterisk (*) and two asterisks (**) indicated significant difference at 0.05 level and 0.01 level, respectively.

### Health risk assessment under different Zn treatments and Cu treatments

**Figure 5:**
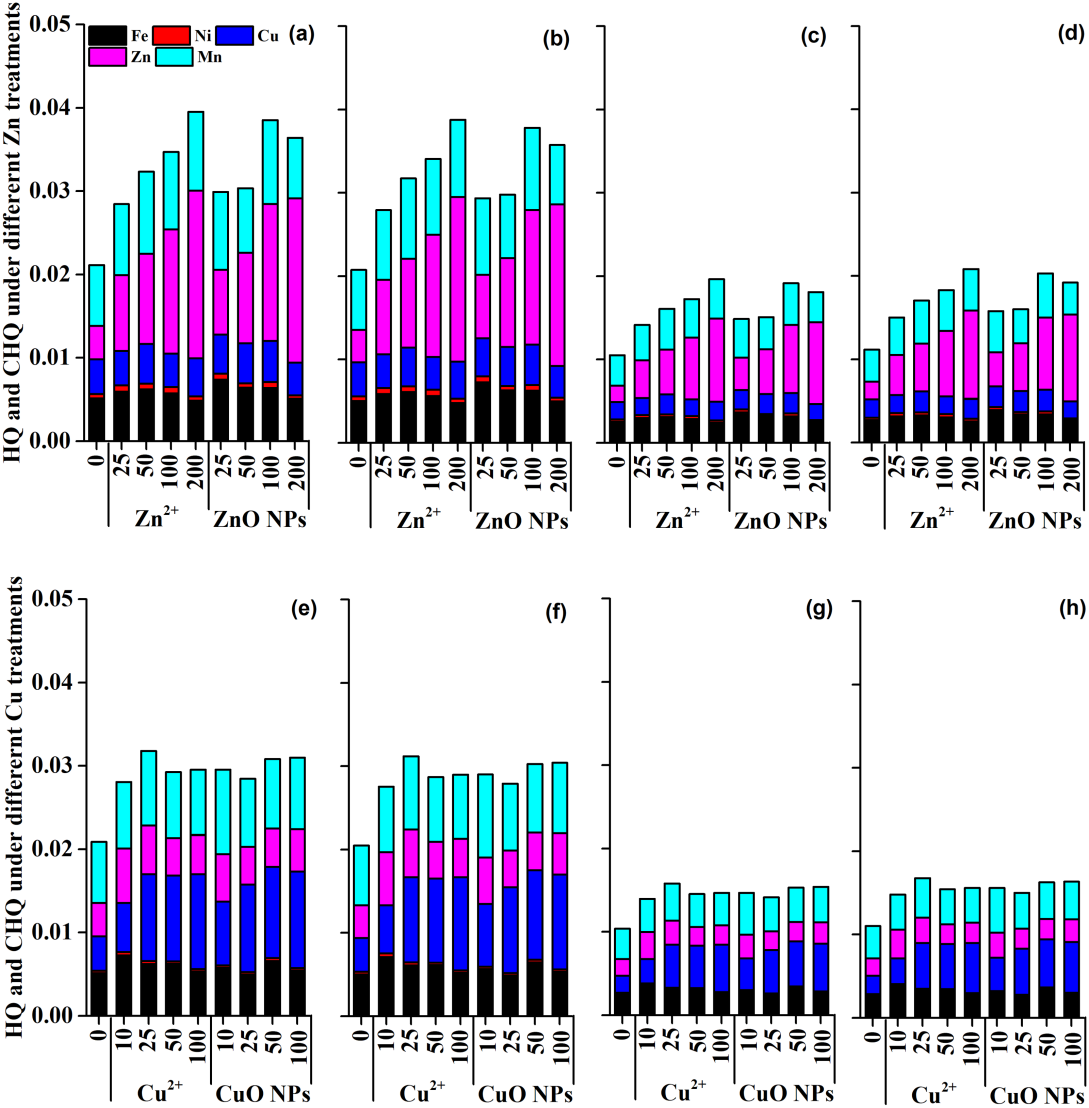
The hazard quotient (HQ) and cumulative hazard quotient (CHQ) of metal elements under different Zn and Cu treatments. The figure (A), (B), (C) and (D) represent the HQ and CHQ of metal elements for adult male, adult female, young child male, and young child female under Zn treatments, respectively. The figure (E), (F), (G) and (H) represent the HQ and CHQ of metal elements for adult male, adult female, young child male, and young child female under Cu treatments, respectively.

Assessing the effect of Zn and Cu on the micronutrient content in the seasonal vegetable *Medicago polymorpha* L. has important implications for human health. Zn and Cu treatments enhanced the absorption of Zn, Cu, Ni, Fe, and Mn in the edible part of *Medicago polymorpha* L. Fortunately, the estimated daily intake (EDI) of metal elements by consuming the ZnO and CuO NPs treated vegetable was quite lower than the oral reference dose (Table S1). The hazard quotient (HQ) and cumulative hazard quotient of metal elements (CHQ) under different Zn treatments and Cu treatments were far less than 1 (Fig. 5). These results indicated that there was no risk of non-carcinogenic effects on the human body exposed to the Zn-treated and Cu-treated plants.

## Discussion

### Response of biomass production to Zn and Cu treatments

Biomass accumulation is determined by photosynthetic area, net photosynthetic rate, and duration (Liu et al., 2019). It has been reported that metal oxide NPs could promote biomass production due to the enhanced net photosynthetic rate, total chlorophyll, and decreased leaf senescence (Dimkpa et al., 2017; Wang et al., 2019; Yang et al., 2021). Raliya et al. (2015) found that ZnO NPs could increase the photosynthetic rate of the leaf by enhancing the chlorophyll content in leaves. Wang et al. (2019) reported that CuO NPs at 200 and 400 mg kg^−1^ for 60 days increased the aboveground biomass of lettuce by 16.3–19.1%, which might be attributed to its improvement in the plant photosynthesis system. In the present study, ZnO and CuO NPs increased the FWPP by 5.8–11.8%, and 3.7−8.1%, respectively (Fig. 1). The FWPP was significantly correlated with the accumulation of Fe, Ni, and Mg under Zn and Cu treatments (Table 3). Fe and Mg played an essential role in photosynthesis and the biosynthesis of chlorophyll (Rui et al., 2016; Zhao et al., 2014). Ni was an important element which was related to N metabolism in photosynthetic foliar tissue (Bai, Reilly & Wood, 2006). Therefore, the enhanced absorption of Fe, Mg, and Ni in plants promoted photosynthesis, resulting in an increased biomass production in *Medicago polymorpha* L.

The beneficial effects of Zn and Cu treatments on FWPP was dependent on their concentrations. The metal ionic and metal oxide NPs exhibited higher efficiency for crop growth promotion at low concentrations than at relatively high concentrations (Fig. 1). This was primarily due to the excess application of Zn and Cu which induced negative effects (*e.g.*, oxidative stress) on the growth of *Medicago polymorpha* L (Hong et al., 2015). Du et al. (2019) reported that Zn^2+^ and ZnO NPs in the concentration range of 20–200 mg kg^−1^ produced the higher aboveground biomass than for the control, and the aboveground biomass significantly decreased when the treatment concentration increased up to 1,000 mg kg^−1^. Raliya et al. (2015) also reported that the biomass increased by 40.7% under ZnO NPs at 100 mg kg^−1^ than for the control, but decreased by 10.9% when the ZnO NPs at 1,000 mg kg^−1^. Regarding of CuO NPs, at a relatively lower concentration (1 mg kg^−1^) in soil, CuO NPs increased the fresh weight of root, stem, and leaf of tomato by 198.5%, 106.2% and 77.5%, and dry weight of root, stem, and leaf by 462.4%, 154.5% and 242.8% compared to the control, respectively. At a relatively higher concentration, Apodaca et al. (2017) reported that CuO NPs at 50 and 100 mg kg^−1^ did not affect the leaf, stem, and root biomass of kidney beans. Wang et al. (2020) suggested that CuO NPs or Cu^2+^ at 75 150, 300, and 600 mg kg^−1^ did not cause significant changes in fresh weight, plant height, water content, and the chlorophyll content of green onion compared with the control. However, Wang et al. (2019) reported that CuO NPs at 200–400 mg kg^−1^ increased the shoot biomass of lettuce by 16.3–19.1%. This indicated that different species have different sensitivity to metal oxide NPs, and the response of species to metal oxide NPs may be dependent on their concentrations. In addition, there was no significant difference in FWPP of *Medicago polymorpha* L. between ionic metal treatments and metal oxide nanoparticles treatments (*p* = 0.51) (Table 2 and Fig. 1). Ebbs et al. (2016) reported that the effect of metal oxide (ZnO, CuO, CeO_2_) NPs treatments was not different from that of ionic metal (Zn^2+^, Cu^2+^, and Ce^4+^) treatments. This indicated that, at the tested concentrations, ZnO and CuO NPs application promoted the biomass production of *Medicago polymorpha* L., and the concentration tested were not toxic for biomass production of *Medicago polymorpha* L. compared with Zn^2+^ and Cu^2+^. Previous studies demonstrated that the toxicological effects of metal oxide NPs were dependent on the metal oxide particle size and treatment concentrations (Deng et al., 2020; Deng et al., 2022). For example, Deng et al. (2020) reported that CuO NPs (75–600 mg kg^−1^) caused more physiological impairments compared with bulk CuO and Cu^2+^ in bok choy. However, in the present study, the treatment concentrations (10–100 mg kg^−1^) were lower than the treatment concentrations reported by Deng et al. (2020). In general, the toxicological effects of CuO NPs increased with the increasing Cu concentration in plant organs (Deng et al., 2020). In our study, there was no significant difference in Cu concentration between CuO NPs and Cu^2+^ at high treatment concentrations (25-100 mg kg^−1^). This may partially explain why there was no significant difference in biomass production between CuO NPs treatments and Cu^2+^ treatments.

### Response of macronutrients uptake to Zn and Cu treatments

N, P and K are the macronutrients required for plant growth. In the present study, low concentrations of Zn^2+^ and ZnO NPs did not affect N accumulation, but high concentrations of Zn^2+^ and ZnO NPs decreased N accumulation (Fig. 2A). Similar results were reported for sorghum, where the application of Zn^2+^ and ZnO NPs decreased aboveground N uptake (Dimkpa et al., 2017). Aziz, Shah & Rashid (2019) reported that ZnO NPs at 1.4−3.6 mg kg^−1^ did not increase N uptake of herbage. Conversely, Yang et al. (2021) reported that the ZnO NPs at 25 and 100 mg kg^−1^ significantly enhanced the aboveground N uptake of rice, primarily due to the increased sink capacity of rice plants (aboveground dry weight). In the present study, both the Zn^2+^ and ZnO NPs increased the aboveground dry weight of *Medicago polymorpha* L. plants (Fig. 1A). This indicated that other factors might affect the aboveground N uptake. At a relatively high concentration (50–500 mg kg^−1^), ZnO NPs decreased the microbial biomass and diversity in the soil (Ge, Schimel & Holden, 2011), and thereby decreased the soil mineral N availability in the soil, decreasing the growth and N uptake of plants (Shah et al., 2021). Cu^2+^ at 100 mg kg^−1^ and CuO NPs at 50–100 mg kg^−1^ substantially decreased the N accumulation of *Medicago polymorpha* L. plants, which was similar to the results of the Zn treatments (Fig. 2F). Zuverza-Mena et al. (2015) reported that CuO NPs 20–80 mg kg^−1^ did not affect the root length, but they decreased the absorption of macronutrients. Xu et al. (2015) reported that CuO NPs at 100-1,000 mg kg^−1^ decreased the soil microbial biomass, the composition and diversity of the paddy soil microbial community, and reduced the urease activity in the soil. This negative effect on soil microorganisms and urease at high concentration of CuO NPs might reduce the available N content in the soil, causing a reduction in growth and the N uptake of plants.

Zn treatment concentrations significantly enhanced the P accumulation of *Medicago polymorpha* L. plants (*p* < 0.001), and P accumulation showed decreasing trend with the increasing Zn treatment concentration (Fig. 2B). Raliya & Tarafdar (2013) found that the application of ZnO NPs at 10 mg L^−1^ increased the P nutrient-mobilizing enzyme, and therefore, increased the P uptake of cluster bean. Their subsequent research also found that ZnO NPs at 10 mg L^−1^ increased the activity of P-solubilizing enzymes, and thereby increasing the P uptake of mung bean (Raliya, Tarafdar & Biswas, 2016). However, the high concentration of ZnO NPs may be toxic to soil microorganisms and decrease soil enzyme activity (Ge, Schimel & Holden, 2011). Chai et al. (2015) investigated the effects of metal oxide NPs on functional bacteria and metabolic profiles in agricultural soil. They reported that ZnO NPs at 1,000 mg kg^−1^ decreased the P-solubilizing bacteria and inhibited the enzymatic activities. This adverse effect may impact P uptake of plants.

Cu treatment concentrations significantly enhanced the P accumulation of *Medicago polymorpha* L (*p* <0.001) (Fig. 2G). Our results were similar with the effects reported by Hong et al. (2015). They found that CuO NPs increased shoot P uptake of alfalfa. They suggested that CuO NPs down-regulated Pht1 (a transporter responsible for P acquisition from soil solution) and up-regulated Pht2 (a transporter responsible for P translocation to the above-ground plant organs), promoting P acquisition and translocation (Hong et al., 2015). In addition, metal oxide NPs may adsorb phosphate ions, modify the P speciation, and cause stress in the rhizosphere. This may result in enhanced root exudation and acidification, leading to the improved P availability and increased P absorption of plants (Zahra et al., 2015). On the contrary, CuO NPs and Cu^2+^ decreased the shoot P content of lettuce (Hong et al., 2015). Zuverza-Mena et al. (2015) also reported that CuO NPs and Cu^2+^ at 20 and 80 mg kg^−1^ reduced the P accumulation of cilantro, indicating that the effects of CuO NPs or Cu^2+^ on P uptake of plants were species-dependent.

Compared with the control, both Zn and Cu treatment concentrations (*p* < 0.001 and *p* = 0.002) significantly increased the K accumulation of plants except for the ZnO NPs treatment at 200 mg kg^−1^ (Figs. 2C and 2H). This indicated that high concentration of ZnO NPs had negative effects on K uptake of *Medicago polymorpha* L and the high concentration of ZnO NPs may reduce the number of K-solubilizing bacteria in the soil (Chai et al., 2015). CuO NPs at 10 mg kg^−1^ produced the highest K accumulation, with 6.1% higher than for CuO NPs at 100 mg kg^−1^ (Fig. 2H). Wang et al. (2020) reported that higher Cu^2+^ acquisition could reduce K accumulation in scallion plants. Deng et al. (2020) reported that higher concentration of CuO NPs (600 mg kg^−1^) had a negative effect on the K uptake compared with the lower concentration of CuO NPs. This was because the high concentration of CuO NPs induced a more complexation with K^+^, reducing their availability for plants uptake (Deng et al., 2020).

### Response of micronutrients uptake to Zn and Cu treatments

Micronutrients (Fe, Ni, Zn, Cu, and Mn) were the important nutrient element for plant growth. Zn and Cu treatments increased the accumulation of Fe (Fig. 3A). However, Dimkpa et al. (2017) reported that ZnO NPs at 6 mg kg^−1^ significantly inhibited the Fe absorption of sorghum. Nair & Chung (2017) reported that ZnO NPs at 20–200 mg kg^−1^ had no significant effect on the Fe concentration of *Arabidopsis*. This indicated that the response of Fe accumulation to ZnO NPs varied in different crops. There was a significantly positive correlation between the accumulation of Fe and Mg and aboveground fresh weight under Zn treatments (*r* = 0.89, *p* < 0.01 and *r* = 0.98, *p* < 0.01) and Cu treatments (*r* = 0.84, *p* < 0.01 and *r* = 0.76, *p* <0.05) (Table 3). Fe played an essential role in many physiological processes, including photosynthesis and the biosynthesis of chlorophyll (Rui et al., 2016). Mg is essential in plants because it is the core of the chlorophyll molecule (Zhao et al., 2014). The increased Fe and Mg uptake under Zn treatments and Cu treatments enhanced the photosynthesis and chlorophyll content and promoted biomass production. As expected, Zn treatments significantly increased the accumulation of Zn (*p* < 0.001), and Cu treatments significantly increased the accumulation of Cu (*p* < 0.001). However, high concentrations of Zn (200 mg kg^−1^) decreased the uptake of Cu compared with the low treatment concentration (25 mg kg^−1^). Similarly, high concentrations of Cu (100 mg kg^−1^) decreased the uptake of Zn compared with the low treatment concentration (25 mg kg^−1^) (Fig. 3D and Fig. 3H). Zhao et al. (2014) reported that the uptake of Cu was decreased by ZnO NPs at 400–800 mg kg^−1^, because Zn^2+^ and Cu^2+^ competed for the same transporter. High concentrations of Zn and Cu decreased the uptake of Ni compared with the low treatment concentration (Fig. 3B and Fig. 3G). Our results were consistent with the findings reported by Zuverza-Mena et al. (2015). Zn^2+^ and Cu^2+^ inhibited Ni^2+^ absorption and translocation in soybean because they shared the same transporter (Zuverza-Mena et al., 2015). The uptake of Mn was increased by the application of Zn and Cu (Fig. 3E and Fig. 3J). Zhao et al. (2014) reported that the absorption of Mn in cucumber plants was increased by ZnO NPs at 400 mg kg^−1^. Zuverza-Mena et al. (2015) found that CuO NPs at 40 mg kg^−1^ increased the uptake of Mn in cilantro.

### Health risk assessment under Zn and Cu treatments

Metal micronutrients (Zn, Cu, Ni, Fe, and Mn) are essential elements for animals and human beings. However, excessive metal elements intake may cause toxic effects (Rajput et al., 2020). Therefore, the assessment of ZnO and CuO NPs on the micronutrient content in the seasonal vegetable *Medicago polymorpha* L. is essential for a better understanding of food safety and human health. Previous studies had assessed the potential non-carcinogenic health risk of metal NPs and metallic element (Natasha et al., 2020; Singh & Kumar, 2020; Shen et al., 2022; Wang et al., 2022). In the present study, the EDI, HQ, and CHQ were used to assess the potential non-carcinogenic health risk of *Medicago polymorpha* L. treated with ZnO and CuO NPs According to our results, ZnO and CuO NPs enhanced the absorption of Zn, Cu, Ni, Fe, and Mn to a certain extent in the edible part of *Medicago polymorpha* L. Fortunately, the EDI was lower than the oral reference dose, and the HQ and CHQ under different Zn treatments and Cu treatments were far below 1 (Fig. 5). Therefore, ZnO and CuO NPs at test concentrations improved the Zn and Cu nutrition in vegetables without causing a non-carcinogenic health risk to human body. Zn and Cu were essential microelements required for plant and human development (Rajput et al., 2018; Tarafdar et al., 2014). Zn played significant roles in a wide variety of metabolic processes, including carbohydrate, lipid, nucleic acid, and protein synthesis as well as their degradation (Rehman et al. , 2012). Cu was also widely distributed in plant tissues and was an essential micronutrient for growth and involved in many physiological processes (Rajput et al., 2018). In addition, other micronutrients, including Fe, Ni, and Mn, increased to a certain extent were improved (Fig. 3). The improved micronutrients not only promoted the growth of *Medicago polymorpha* L., but also improved the microelement nutrition in the edible parts of vegetables. The enriched nutrients in the edible parts of *Medicago polymorpha* L. may be beneficial to improving trace nutrients of human body.

## Conclusion

ZnO and CuO NPs increased biomass production of *Medicago polymorpha* L. in a concentration-dependent fashion. Both ZnO and CuO NPs improved the absorption of macronutrients (P, K, Mg, and Ca) and micronutrients absorption (Fe, Ni, Cu, Zn, and Mn). Comprehensive analysis showed the optimum concentrations were 25 mg kg^−1^ for ZnO NPs and 10 mg kg^−1^ for CuO NPs. The EDI, HQ, and CHQ were further calculated to assess the non-carcinogenic health risk of consuming the *Medicago polymorpha* L. treated with ZnO and CuO NPs. Our results showed that the enhanced metal nutrients in *Medicago polymorpha* L. did not cause any non-carcinogenic health risk to human body. Therefore, our results indicated that the low concentration of ZnO and CuO NPs could enhance growth performance and improve the micronutrient absorption of *Medicago polymorpha* L. without posing any non-carcinogenic health risk to the human body. Taken together, according to growth analysis and a human health risk assessment, ZnO and CuO NPs were considered as a safe nano fertilizer for *Medicago polymorpha* L. production. However, the effect of ZnO and CuO NPs was impacted by many factors, including soil properties and biological effects (Watson et al., 2015). Therefore, further investigation as to the different soil environment conditions are needed to study the effect of ZnO and CuO NPs in M*edicago polymorpha* L.

##  Supplemental Information

10.7717/peerj.14038/supp-1 Supplemental Information 1 Raw data

10.7717/peerj.14038/supp-2 Supplemental Information 2 The concentration of macronutrients and micronutrients of Medicago Polymorpha L. plants under different Zn and Cu treatments

10.7717/peerj.14038/supp-3 Supplemental Information 3 The estimated daily intake (EDI) of metal elements under different Zn and Cu treatments *The data of oral reference dose (RfD) was referred from the studies of Kicinska, Glichowska & Mamak (2019), Shen et al. (2022), Zhu et al. (2011) and Wang et al. (2022).

## Additional Information and Declarations

### Data Availability

The following information was supplied regarding data availability: The raw measurements are available in the Supplementary Files.
