# Supplementary material for: Effect of ZnO and CuO nanoparticles on the growth, nutrient absorption, and potential health risk of the seasonal vegetable Medicago polymorpha L
Source: PeerJ. 2022 Sep 21;10:e14038. doi: 10.7717/peerj.14038 (PMC9508880; doi:10.7717/peerj.14038)
Supplement: Supplemental Information 3 — *The data of oral reference dose (RfD) was referred from the studies of Kicinska, Glichowska & Mamak (2019), Shen et al. (2022), Zhu et al. (2011) and Wang et al. (2022). [file peerj-10-14038-s003.docx]

| Treatment concentration | | Fe (ug kg^-1^ d^-1^) | | | | Ni (ug kg^-1^ d^-1^) | | | | Cu (ug kg^-1^ d^-1^) | | | | Zn (ug kg^-1^ d^-1^) | | | | Mn (ug kg^-1^ d^-1^) | | | |
| --- | --- | --- | --- | --- | --- | --- | --- | --- | --- | --- | --- | --- | --- | --- | --- | --- | --- | --- | --- | --- | --- |
|  |  | Adult male | Adult female | Yong child male | Yong child female | Adult male | Adult female | Yong child male | Yong child female | Adult male | Adult female | Yong child male | Yong child female | Adult male | Adult female | Yong child male | Yong child female | Adult male | Adult female | Yong child male | Yong child female |
|  | 0 | 3.58 | 3.51 | 1.78 | 1.89 | 0.01 | 0.01 | 0.00 | 0.01 | 0.15 | 0.15 | 0.08 | 0.08 | 1.20 | 1.17 | 0.59 | 0.63 | 1.76 | 1.68 | 0.85 | 0.90 |
| Zn^2+^ | 25 | 4.29 | 4.21 | 2.13 | 2.26 | 0.02 | 0.01 | 0.01 | 0.01 | 0.16 | 0.16 | 0.08 | 0.08 | 2.80 | 2.75 | 1.39 | 1.48 | 2.10 | 2.06 | 1.05 | 1.11 |
|  | 50 | 4.28 | 4.20 | 2.13 | 2.26 | 0.01 | 0.01 | 0.01 | 0.01 | 0.18 | 0.18 | 0.09 | 0.09 | 3.16 | 3.10 | 1.57 | 1.67 | 2.37 | 2.33 | 1.18 | 1.25 |
|  | 100 | 3.73 | 3.66 | 1.85 | 1.97 | 0.01 | 0.01 | 0.01 | 0.01 | 0.15 | 0.15 | 0.07 | 0.08 | 4.31 | 4.23 | 2.14 | 2.27 | 2.14 | 2.10 | 1.06 | 1.13 |
|  | 200 | 3.34 | 3.28 | 1.66 | 1.76 | 0.01 | 0.01 | 0.01 | 0.01 | 0.16 | 0.15 | 0.08 | 0.08 | 6.25 | 6.13 | 3.10 | 3.30 | 2.37 | 2.32 | 1.18 | 1.25 |
| ZnO NPs | 25 | 5.24 | 5.14 | 2.60 | 2.76 | 0.01 | 0.01 | 0.01 | 0.01 | 0.17 | 0.17 | 0.08 | 0.09 | 2.41 | 2.37 | 1.20 | 1.27 | 2.24 | 2.20 | 1.11 | 1.18 |
|  | 50 | 4.69 | 4.61 | 2.33 | 2.48 | 0.01 | 0.01 | 0.01 | 0.01 | 0.18 | 0.17 | 0.09 | 0.09 | 3.25 | 3.19 | 1.61 | 1.71 | 2.05 | 2.02 | 1.02 | 1.08 |
|  | 100 | 4.36 | 4.27 | 2.16 | 2.30 | 0.01 | 0.01 | 0.01 | 0.01 | 0.19 | 0.19 | 0.09 | 0.10 | 4.63 | 4.55 | 2.30 | 2.44 | 2.36 | 2.32 | 1.17 | 1.25 |
|  | 200 | 3.45 | 3.39 | 1.72 | 1.82 | 0.01 | 0.01 | 0.00 | 0.01 | 0.15 | 0.14 | 0.07 | 0.08 | 6.25 | 6.13 | 3.11 | 3.30 | 1.80 | 1.77 | 0.89 | 0.95 |
|  | 0 | 3.58 | 3.51 | 1.78 | 1.89 | 0.01 | 0.01 | 0.00 | 0.01 | 0.15 | 0.15 | 0.08 | 0.08 | 1.20 | 1.17 | 0.59 | 0.63 | 1.76 | 1.68 | 0.85 | 0.90 |
| Cu^2+^ | 10 | 5.07 | 4.97 | 2.52 | 2.67 | 0.02 | 0.02 | 0.01 | 0.01 | 0.23 | 0.23 | 0.12 | 0.12 | 1.91 | 1.87 | 0.95 | 1.01 | 1.97 | 1.93 | 0.98 | 1.04 |
|  | 25 | 4.35 | 4.27 | 2.16 | 2.29 | 0.01 | 0.01 | 0.01 | 0.01 | 0.38 | 0.37 | 0.19 | 0.20 | 1.67 | 1.63 | 0.83 | 0.88 | 2.05 | 2.01 | 1.02 | 1.08 |
|  | 50 | 4.38 | 4.30 | 2.18 | 2.31 | 0.01 | 0.01 | 0.01 | 0.01 | 0.38 | 0.37 | 0.19 | 0.20 | 1.34 | 1.31 | 0.67 | 0.71 | 1.88 | 1.84 | 0.93 | 0.99 |
|  | 100 | 3.74 | 3.67 | 1.86 | 1.97 | 0.01 | 0.01 | 0.00 | 0.01 | 0.42 | 0.41 | 0.21 | 0.22 | 1.39 | 1.36 | 0.69 | 0.73 | 1.91 | 1.87 | 0.95 | 1.01 |
| CuO NPs | 10 | 4.05 | 3.97 | 2.01 | 2.14 | 0.01 | 0.01 | 0.01 | 0.01 | 0.28 | 0.27 | 0.14 | 0.15 | 1.66 | 1.63 | 0.83 | 0.88 | 2.40 | 2.35 | 1.19 | 1.26 |
|  | 25 | 3.49 | 3.43 | 1.74 | 1.84 | 0.01 | 0.01 | 0.01 | 0.01 | 0.38 | 0.38 | 0.19 | 0.20 | 1.41 | 1.39 | 0.70 | 0.75 | 2.02 | 1.98 | 1.00 | 1.07 |
|  | 50 | 4.60 | 4.51 | 2.28 | 2.42 | 0.01 | 0.01 | 0.01 | 0.01 | 0.42 | 0.41 | 0.21 | 0.22 | 1.40 | 1.38 | 0.70 | 0.74 | 1.99 | 1.95 | 0.99 | 1.05 |
|  | 100 | 3.82 | 3.74 | 1.90 | 2.01 | 0.01 | 0.01 | 0.01 | 0.01 | 0.42 | 0.41 | 0.21 | 0.22 | 1.44 | 1.42 | 0.72 | 0.76 | 1.97 | 1.93 | 0.98 | 1.04 |
| RfD* | | 700 | | | | 20 | | | | 37 | | | | 300 | | | | 240 | | | |
